# Plasma Calcitonin Gene-Related Peptide: A Potential Biomarker for Diagnosis and Therapeutic Responses in Pediatric Migraine

**DOI:** 10.3389/fneur.2019.00010

**Published:** 2019-01-24

**Authors:** Pi-Chuan Fan, Ping-Hung Kuo, Ming Tatt Lee, Shu-Hui Chang, Lih-Chu Chiou

**Affiliations:** ^1^Department of Pediatrics, National Taiwan University Hospital, Taipei, Taiwan; ^2^Clinical Center for Neuroscience and Behavior, National Taiwan University Hospital, Taipei, Taiwan; ^3^Department of Pediatrics, College of Medicine, National Taiwan University, Taipei, Taiwan; ^4^Graduate Institute of Pharmacology, College of Medicine, National Taiwan University, Taipei, Taiwan; ^5^Department of Internal Medicine, National Taiwan University Hospital, Taipei, Taiwan; ^6^Graduate Institute of Brain and Mind Sciences, College of Medicine, National Taiwan University, Taipei, Taiwan; ^7^Faculty of Pharmaceutical Sciences, UCSI University, Kuala Lumpur, Malaysia; ^8^Department of Epidemiology, College of Public Health, National Taiwan University, Taipei, Taiwan; ^9^Graduate Institute of Acupuncture Sciences, China Medical University, Taichung, Taiwan

**Keywords:** pediatric migraine, calcitonin gene-related peptide, plasma biomarker, anti-migraine drugs, topiramate

## Abstract

**Background:** Plasma calcitonin gene-related peptide (CGRP) plays a key role in the migraine pathophysiology. This study aimed to investigate its role in predicting diagnosis and outcome of pharmacotherapy in pediatric migraine.

**Methods:** We prospectively recruited 120 subjects, who never took migraine-preventive agents in a pediatric clinic, including 68 patients with migraine, 30 with non-migraine headache (NM), and 22 non-headache (NH) age-matched controls. Short-term therapeutic response was measured for at least 2 weeks after the start of therapy. Responders were defined with >50% headache reduction. Plasma CGRP concentrations were measured by ELISA.

**Results:** In the migraine group, more patients required acute therapy, as compared to the NM group (62/68, 91% vs. 5/30, 15%, *p* = 0.001). The mean plasma CGRP level in migraineurs either during (291 ± 60 pg/ml) or between (240 ± 48) attacks was higher than in NM patients (51 ± 5 pg/ml, *p* = 0.006 and 0.018, respectively) and NH controls (53 ± 6 pg/ml, *p* = 0.016 and 0.045, respectively). Forty-seven patients (69%) needed preventive treatments and had higher plasma CGRP levels (364 ± 62 pg/ml, *n* = 47) than those not (183 ± 54 pg/ml, *n* = 21) (*p* = 0.031). Topiramate responders had higher plasma CGRP levels than non-responders (437 ± 131 pg/ml, *n* = 14 vs. 67 ± 19 pg/ml, *n* = 6, *p* = 0.021). Survival curves of plasma CGRP levels also showed those with higher CGRP levels responded better to topiramate. Differences were not found in the other preventives.

**Conclusion:** The plasma CGRP level can differentiate migraine from non-migraine headache. It may also serve as a reference for the therapeutic strategy since it is higher in patients requiring migraine prevention and responsive to short-term topiramate treatment. These results are clinically significant, especially for the young children who cannot clearly describe their headache symptoms and may provide new insights into the clinical practice for the diagnosis and treatment of pediatric migraine.

## Introduction

Migraine, characterized by attacks of severe throbbing headaches with sensory sensitivity to light, sound, and head movement (1), is among the most common disorders and remains one of the leading causes of disability (2, 3). The principles of the pharmacologic treatment consist of acute symptomatic and prophylactic therapies (4). The former is intent on relieving or ameliorating the symptoms of an acute attack while the latter aims to decrease the attack frequency and pain severity through a daily intake of medication for a certain time period (5). The migraine associations such as abdominal pain, limb pain, and motion sickness are common in children (6) but less in adult. Although, it is well-known that medications that are efficacious for adults cannot be used routinely for younger patients (7), there are limited reports on the efficacy and safety of migraine medications in the pediatric group. Only a few randomized placebo-controlled clinical trials for both acute and prophylactic drugs have been conducted in pediatric patients. Biomarkers that can guide the choice of anti-migraine drugs are exceedingly important in the clinical setting, especially for young children who cannot clearly describe their symptoms.

Calcitonin gene-related peptide (CGRP), a 37-amino-acid neuropeptide, is a potent vasodilator for both peripheral and cerebral blood vessels (8, 9). CGRP has been shown to play a causative role in migraine attacks since its level is shown to be elevated during migraine attacks (10). Thus, CGRP is believed to be a potential biomarker of migraine (11–21). Pre-clinical studies have shown that trigeminal activation can induce CGRP release from peri-vascular nerve endings (22–24), resulting in pia vessel dilatation, leading to migraine (17). Moreover, CGRP antagonists can reduce cortical spreading depression, which is believed to be an important pathogenic manifestation of migraine with aura (18, 20, 21), in animal models of migraine. Clinically, baseline CGRP levels in adult patients are considerably higher than those in healthy controls (15). During migraine attacks, plasma CGRP levels are elevated in both adult (13) and pediatric (12, 19) patients, and their changes correlate with headache intensities (15, 19). Several clinical pharmacological studies also support the notion that CGRP plays a causative role in migraine. First, intravenous infusion of CGRP produces a migraine-like headache in volunteers (14). Second, a CGRP receptor antagonist, olcegepant (BIBN4096BS), is effective in treating acute migraine attacks (16) and anti-CGRP or anti-CGRP receptor monoclonal antibodies are approved for migraine prevention (25, 26). Third, triptans, clinically effective anti-migraine agents, can also reduce CGRP levels in cats and humans (11).

We have previously proven the diagnostic value of the plasma CGRP level in pediatric migraine, especially during migraine attack (19). The present study further explored whether this biomarker is also useful in guiding the choice of anti-migraine drugs by investigating the correlation between plasma CGRP levels and the responses of acute and preventive pharmacotherapeutics of pediatric migraine.

## Methods

### Study Subjects

We prospectively recruited 120 consecutive study subjects at the pediatric outpatient clinic of National Taiwan University Hospital (NTUH), a tertiary medical center in Taiwan. They were aged from 5 to 18 years with 60% (72/120) below 12 years and had never taken any medicine for migraine prevention. There were 68 patients with migraine, 30 patents with non-migraine headache and 22 non-headache control subjects. The diagnosis of pediatric migraine was based on the criteria of the International Classification of Headache Disorders, 3rd Edition (Beta Version) (27). The exclusion criteria were: herbal drug intake, intra-cranial mass, mental retardation, congenital, or chromosomal anomaly, other major organ diseases, and pregnancy. The study protocol had been approved by the Scientific Ethical Committee of NTUH and the informed consent had been signed by their parents and the study subjects older than 7 years.

**Figure 1:**
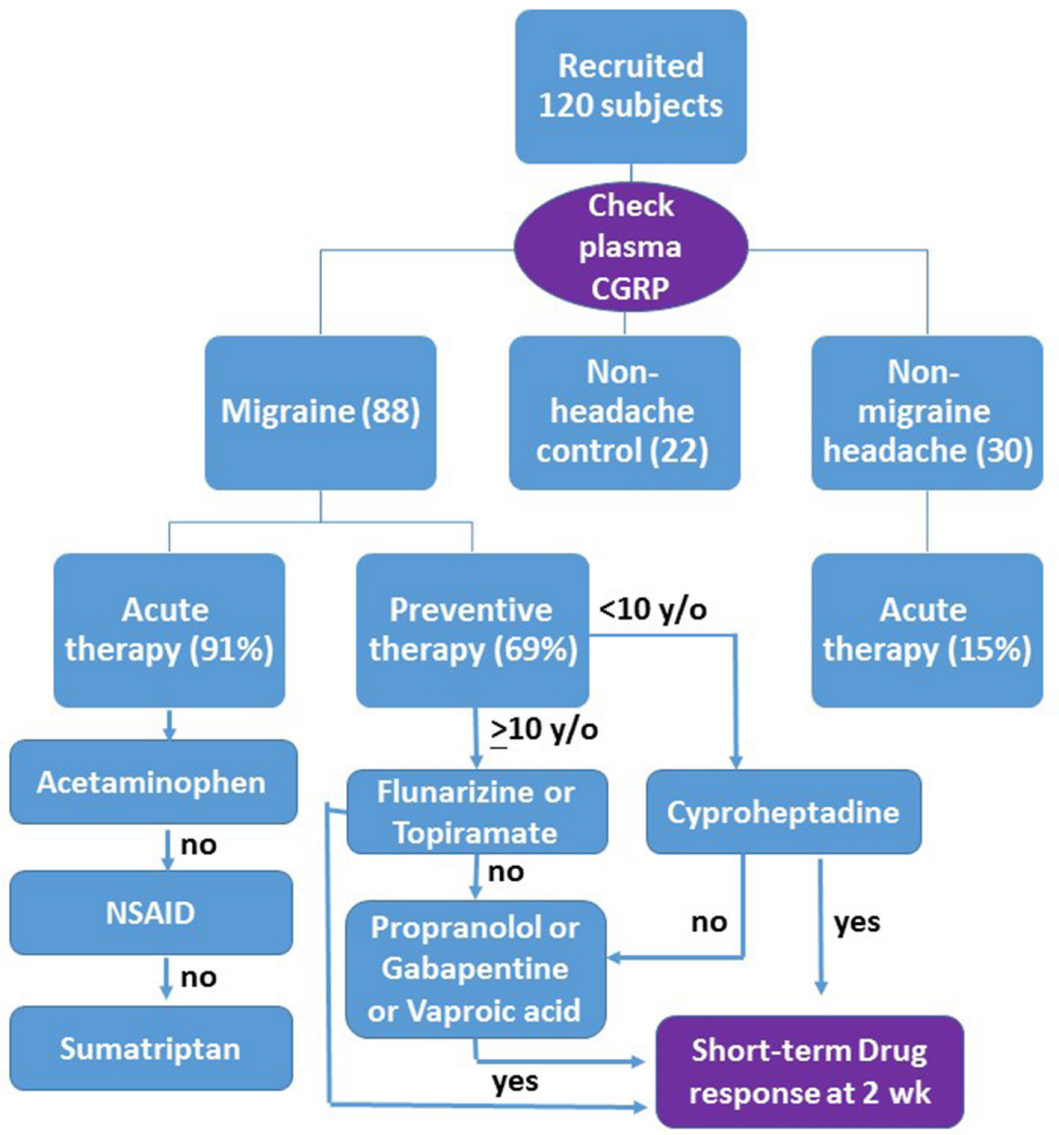
Flow chart of the design as described in the text. Those patients who have more than 50% headache reduction are responsive to the drug (yes), otherwise (no) go to the second line or third line therapy. The short-term drug response is evaluated after at least 2 weeks of drug use.

Patients who were headache-free for at least 24 h before and after blood sampling were classified as the non-attack group. In the attack group, the headache conditions were recorded when blood samples were collected, performed within the first 8 h of the headache episode. Data collected included age, sex, age of onset, electroencephalography (EEG) and the headache diary in which the headache severity, duration, and frequency were recorded. Headache severity was scored in an 11-point scale, with the 0 for non-headache and 10 for the most severe intensity. Patients with the headache severity score >5 were given an acute treatment with acetaminophen (10–15 mg/kg), naproxen (250 mg), or sumatriptan (50 mg) as the first-, second-, and third-line therapies, respectively (flow chart shown in Figure 1). Patients with headache severity score <5 did not receive any drug for acute attack.

Those patients who had more than 4 major headache attacks with headache severity score >5 every month received a preventive treatment with flunarizine (5 mg) or topiramate (50 mg) prescribed randomly as the first-line therapy for patients older than 10 years. Both drugs were approved as anti-migraine drugs by the National Health Insurance in Taiwan. Cyproheptadine (4–6 mg) was prescribed only for patients younger than 10 years in consideration of a possible adverse effect of sleepiness in older ones (Figure 1). Amitriptyline, a tricyclic antidepressant used as first line anti-migraine drug in many countries, is not available in NTUH.

In consideration of the drug compliance in children and the intent to seek for efficient migraine preventives, we measured the short-term therapeutic response for at least 2 weeks after the start of therapy (Figure 1). It was scaled as the headache reduction in either duration or severity >90% (significant), 50–90% (moderate), <50% (minimal), and no effect. Responders were defined as patients with >50% headache reduction. For non-responders, a second-line drug, such as propranolol (20 mg), gabapentin (300 mg), or valproic acid (500–750 mg), was randomly prescribed to replace or to add to the first drug (Figure 1). No more than two drugs were used simultaneously. Additive drug response was recorded in two combined treatments. Adverse effects of treatments were also recorded.

### Plasma CGRP Measurement

Blood (3 ml) sampled from the cubital vein of each patient was put into a 5 ml Lavender tube (BD vacutainer^TM^, Becton Dickenson, Plymouth, UK). Plasma samples were prepared by centrifugation (2,000 rpm for 15 min) of the whole blood and then stored at −80°C. Plasma CGRP levels were measured using a commercial enzyme-linked immuno-sorbent assay (ELISA) kit (SPI-BIO, City, France). The person who conducted the CGRP measurement was blinded to the identity, attack status, and treatment of the study patients.

### Statistical Analysis

Data were expressed as mean ± standard error (SE) unless otherwise specified. All categorical variables were analyzed by chi-square tests, except in small sample sizes (<5), where Fisher's exact test was used. Differences between groups were compared by Student's *t*-test or the Mann-Whitney *U* test, depending on variable distribution by the Kolmogorov-Smirnov test. Differences among groups were compared by ANOVA followed by the Tukey HSD *post-hoc* test. Predictive performance of CGRP levels was evaluated by analyzing the receiver operator characteristic (ROC) curves, from which the sensitivity, specificity, and accuracy of the attack CGRP level in predicting drug responders were estimated. Statistical significance was set at *p* < 0.05. All statistical analyses were performed using SPSS software (version 20.0; SPSS Inc., Chicago, IL).

## Results

Plasma samples were collected from 68 patients with migraine (28 boys) aged 5–18 years (mean age, 11.7 ± 0.4 years), 30 with non-migraine headache (12 boys; 9.6 ± 0.7 years) and 22 non-headache controls (13 boys; 10.1 ± 0.8 years). The mean age of the migraineurs is higher than that of non-migraine headache patients (*p* = 0.018), but not significantly different from the non-headache controls (*p* > 0.05). Among patients with migraine, 31 (34%) were blood-sampled during headache attack and 57 (66%) between attacks. The condition at sampling, either during or between headache attacks, did not correlate with the response to each drug (*p* > 0.05). In the migraine group, 91% (62 out of 68 patients) required acute therapy, which is significantly higher than the ratio (15%, 5/30) in the non-migraine headache group (*p* = 0.001). There were 47 patients (69%) needing preventive treatments. Among them, 39 patients (83%) received monotherapy and 8 (17%) were treated with two combined drugs.

### Responses to Acute Therapy

**Figure 2:**
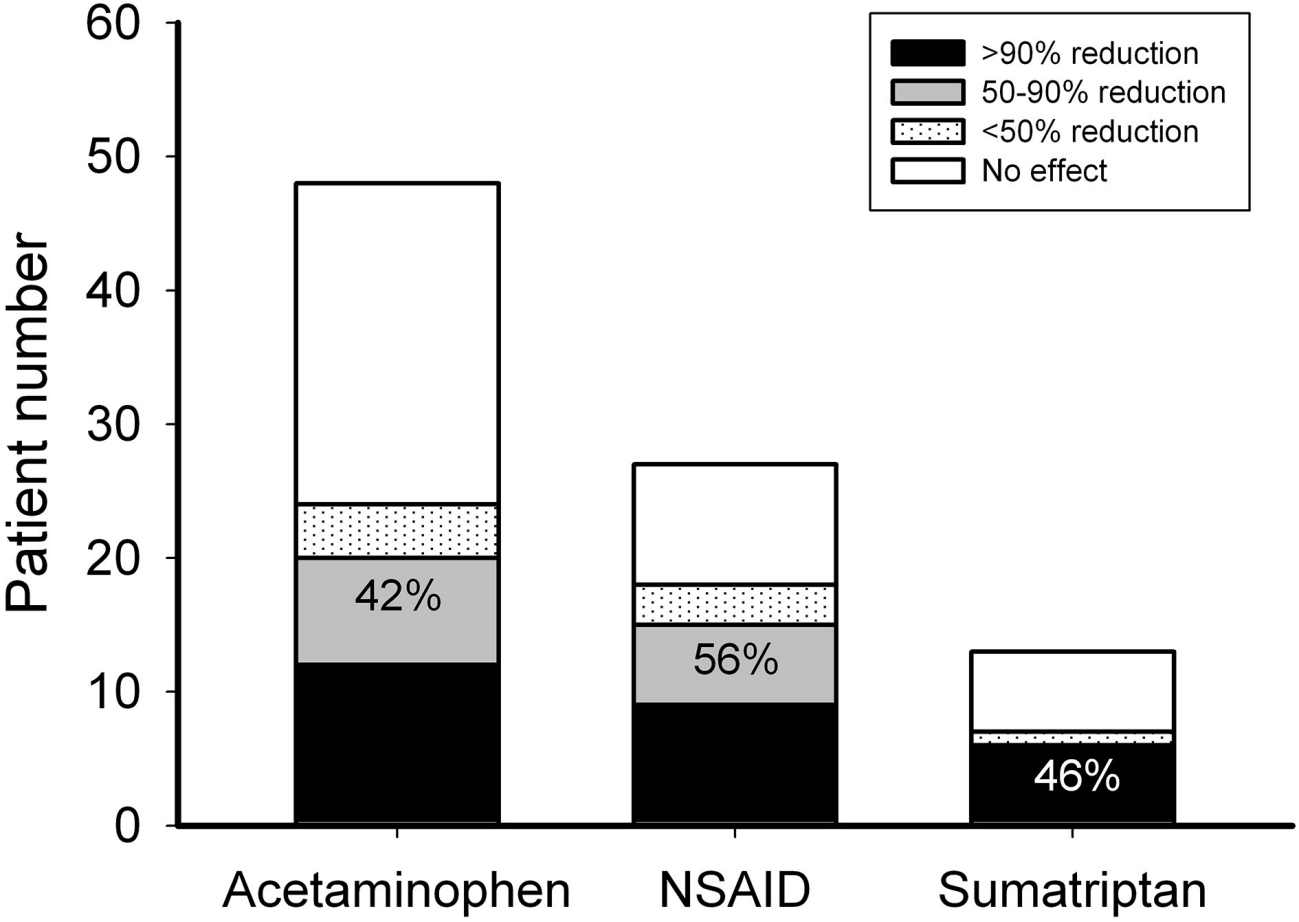
Responsiveness to acute anti-migraine therapy. Patients who needed acute therapy were treated with acetaminophen, naproxen, or sumatriptan as the first-, second-, and third-line choices, respectively. The ordinate is the numbers of patients with 0 (open bar), <50 (dotted bar), 50–90 (gray bar), and >90% (black bar) headache reduction. The number depicted on the bar is the response rate of patients with headache reduction ≥50% (black + gray bars).

In the patients requiring acute therapy, 20 of 48 patients (42%) responded to acetaminophen with headache reduction >50%, while 15 of 27 (56%) and 6 of 13 (46%) patients responded to naproxen and sumatriptan, respectively (Figure 2). There was no adverse effect reported with acetaminophen intake, two (7%) complained of abdominal pain after taking naproxen and four (29%) reported nausea, flushing, numbness, or general weakness after taking sumatriptan.

### Short-Term Responses to Preventive Therapy

**Figure 3:**
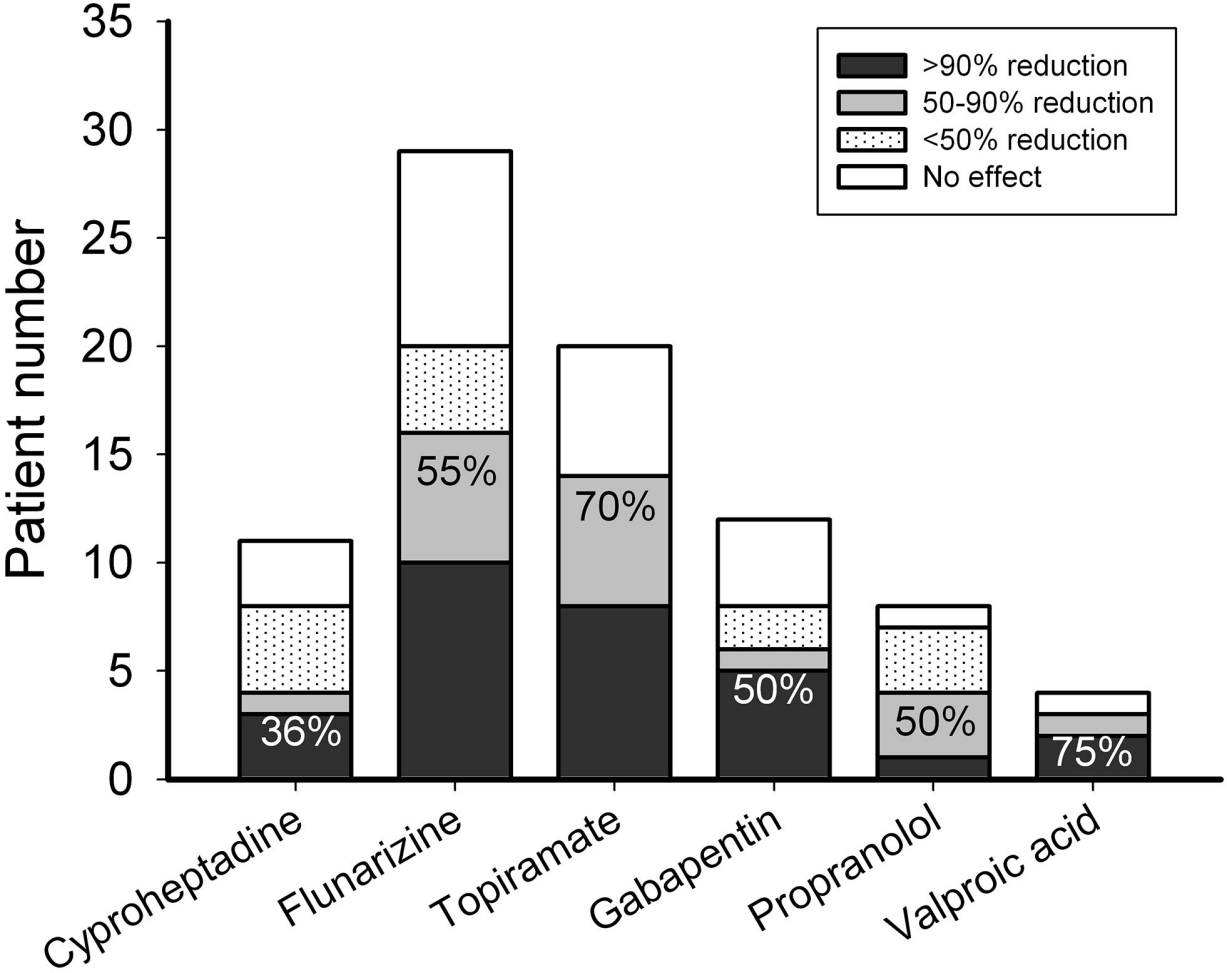
Responsiveness to preventive anti-migraine therapy using cyproheptadine, flunarizine, topiramate, gabapentin, propranolol, or valproic acid. The ordinate is the numbers of patients with 0 (open bar), <50 (dotted bar), 50-90 (gray bar), and >90% (black bar) headache reduction. The number depicted on the bar is the response rate of patients with headache reduction ≥50% (black + gray bars).

Among patients who needed preventive treatment, 4 of 11 (36%) patients responded to cyproheptadine, 16 of 29 (55%) to flunarizine, 14 of 20 (70%) to topiramate, 6 of 12 (50%) to gabapentin, 4 of 8 (50%) to propranolol, and 3 of 4 (75%) to valproic acid (Figure 2). Three patients (10%) treated with flunarizine suffered from dizziness, depression, and skin rash, respectively. Two patients (10%) who took topiramate had paresthesia or vomiting. One (8%) who was on gabapentin had prolonged headache and two (50%) on valproic acid had hair loss or visual hallucination. There was no obvious adverse effect reported with cyproheptadine or propranolol (Figure 3).

### Plasma CGRP and Migraine Treatment

**Figure 4:**
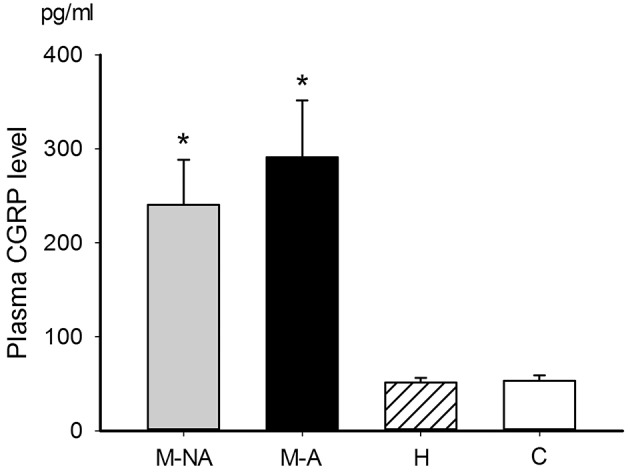
Plasma CGRP levels in different groups. The mean plasma CGRP levels in the migraineurs either during (291 ± 60 pg/ml, *n* = 31, group M-A in black bar) or between (240 ± 48 pg/ml, *n* = 57, group M-NA in gray bar) attacks were higher than the non-migraine headache patients (51 ± 5 pg/ml, *n* = 30, group H) (*p* = 0.006 and 0.018, respectively) and non-headache controls (53 ± 6 pg/ml, *n* = 22, group C) (*p* = 0.016 and 0.045, respectively). The *p*-value among groups, analyzed by ANOVA test (*p* = 0.001), followed by *post-hoc* Tukey HSD. *p < 0.05.

Plasma CGRP levels were significantly different among migraine (during or between attacks), non-migraine headache, and non-headache control groups (ANOVA *p* = 0.001). Mean plasma CGRP levels in migraineurs during and between attacks were 291 ± 60 pg/ml and 240 ± 48 pg/ml, respectively. They were higher than that (51 ± 5 pg/ml, *p* = 0.006 and 0.018, respectively) of patients with non-migraine headache and that (53 ± 6 pg/ml, *p* = 0.016 and 0.045, respectively) of non-headache controls (Figure 4). The plasma CGRP level was not significantly correlated with the sex, EEG abnormality or headache phase (attack or non-attack) in migraineurs (Table 1). There was also no significant difference between patients who needed acute therapy and those who did not. However, the mean plasma CGRP level was higher in patients who required preventive therapy, as compared to those who did not (*p* = 0.031) (Table 1).

**Table 1:** Comparison of plasma CGRP levels between attack and non-attack, normal and abnormal EEG, and responder and non-responder to anti-migraine drugs.

**Conditions**	**CGRP in pg/ml (*****n*****)**		***p*-value^**§**^**
	**Yes**	**No**	
Attack at sampling	298 ± 65 (27)	315 ± 65 (41)	0.866
Abnormality of EEG	296 ± 157 (9)	276 ± 54 (51)	0.89
Responder of acute therapy^a^	295 ± 47 (62)	348 ± 224 (6)	0.751
Responder of preventive therapy^b^	364 ± 62 (47)	183 ± 54 (21)	0.031^*^

*Data are mean ± S.E. with the n number in parentheses*.

a *Acute therapy was administered to abort a single severe attack*.

b *Preventive therapy was taken regularly for frequent attacks*.

§ *The p-value between plasma CGRP levels of the variables in the left columns of conditions, as analyzed by Student's t-test or Mann-Whitney test as described in the text*.

* *p < 0.05*.

**Figure 5:**
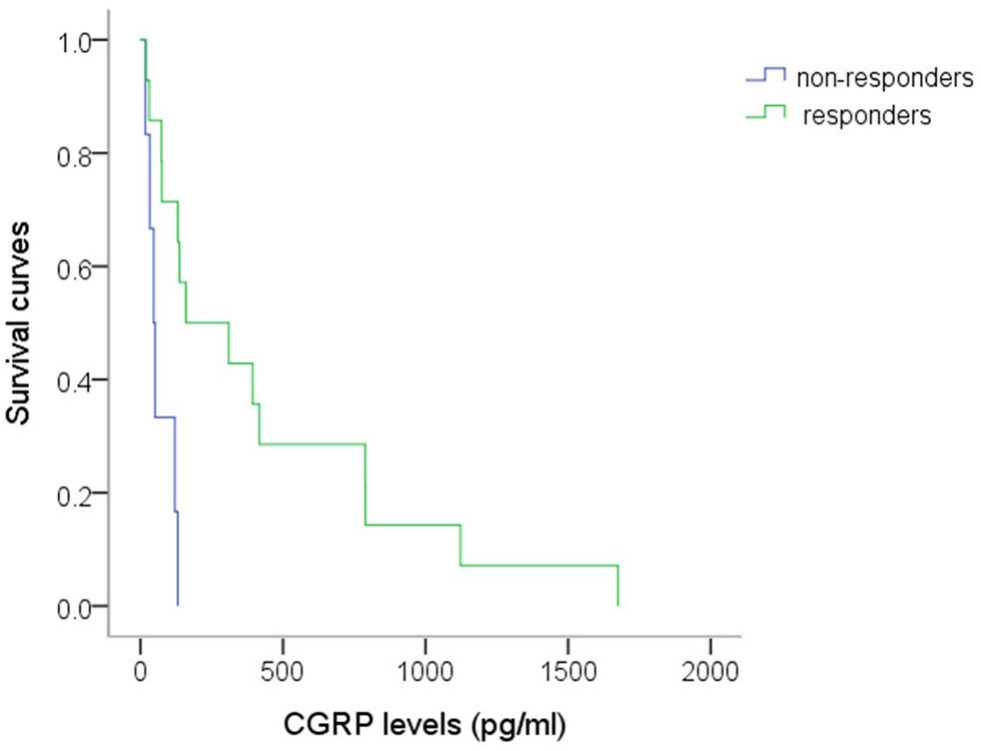
The survival curves of CGRP in responders and non-responders to topiramate. The survival function of CGRP at a given level (x) means the probability that the CGRP for a patient is beyond this specified level (x axis). Distribution of the CGRP levels at t is equal to the probability of the patient's CGRP level ≤ t level. The responders' survival curve at all level of CGRP is always above the non-responders'. Thus, it suggests those patients with higher CGRP levels have a higher probability to respond to topiramate, using Mann-Whitney test, (*p* = 0.021). *n* = 14 vs. 6 (responders vs. non-responders).

Table 2 shows plasma CGRP levels between responders and non-responders in each drug-treatment group. The responders to topiramate had higher CGRP levels (437 ± 131 pg/ml, *n* = 14), as compared to non-responders (67 ± 19 pg/ml, *n* = 6, *p* = 0.021). The empirical survival curves (the distribution) of CGRP between responders and non-responders also showed that the distributions of CGRP between the two groups are very different and those with higher CGRP levels responded better to topiramate (Figure 5). Using a threshold of 62.57 or 125.97 pg/ml, the sensitivity of the plasma CGRP level in predicting topiramate responders was 0.86 and specificity 0.67, or 0.71 and 0.83 (AUC = 0.833), respectively, despite the limitation of small sample size. There was no correlation between the responsiveness and the age, sex, and headache phase (attack or non-attack). However, in all of the other drug-treatment groups, there was no significant difference in CGRP levels between responders and non-responders.

**Table 2:** Comparison of plasma CGRP levels between responders and non-responders to anti-migraine drugs.

**Anti-migraine drug**	**CGRP in pg/ml (*****n*****)**		***p*-value^**§**^**
	**Responders**	**Non-responders**	
**ACUTE THERAPY**^a^			
Acetaminophen	256 ± 44(20)	304 ± 83(28)	0.352
NSAID	309 ± 119(15)	117 ± 44(12)	0.884
Sumatriptan	61 ± 17(6)	397 ± 236(7)	0.317
**PREVENTIVE THERAPY**^b^			
Cyproheptadine	191 ± 52(4)	473 ± 188(8)	0.45
Flunarizine	243 ± 77(17)	466 ± 158(13)	0.219
Topiramate	437 ± 131(14)	67 ± 19(6)	0.021^*^
Gabapentin	421 ± 253(6)	352 ± 265(6)	0.522
Propranolol	418 ± 329(4)	97 ± 24(4)	0.564
Valproate	586 ± 544(3)	74 (1)	0.655

*Data are mean ± S.E with number (n) in parentheses*.

a *Acute therapy was administered to abort a single severe attack*.

b *Preventive therapy was taken regularly for frequent attacks*.

§ *The p-value between responder and non-responder groups, analyzed by Mann-Whitney test*.

* *p < 0.05*.

*ND, not determined*.

## Discussion

Among the pediatric migraineurs recruited in the present study, we found that around 90% required acute anti-migraine therapy and 70% needed preventive therapy. For acute therapy, NSAIDs, even as the second line, had better treatment outcome than acetaminophen. For preventive therapy, the short-term response rate to topiramate (70%) was higher than that of flunarizine (55%) among the first-line monotherapy. One-third of cyproheptadine-treated children were responders. The plasma CGRP levels in migraineurs, either during or between attacks, were significantly higher, as compared to non-migraine headache patients or age-matched non-headache controls. This supports the notion that plasma CGRP is a potential diagnostic biomarker for pediatric migraine (19). Furthermore, a relatively higher plasma CGRP level was noted in those patients who required preventive therapy and are the responders to topiramate. This suggests the potential for the plasma CGRP level to serve as a biomarker guiding the pediatric migraine treatment, which has never been mentioned before.

According to the response rate to acute therapies in the present study, we estimate at least 14% [(1–0.42)^*^(1–0.56)^*^(1–0.46)] of these patients were refractory to current acute pharmacologic therapy. Acetaminophen and NSAIDs have been reported to be both significantly more effective than placebo in pain relief in a double-blind cross-over study of childhood migraine (28). The response rate of acetaminophen (42%) in the present study is relatively lower than that (53%) in another prospective, double-blind, placebo-controlled study involving younger patients (6–12 years old) (29). The results here also show that even as a second-line drug of choice for acute migraine treatment, NSAIDs are better than acetaminophen (response rate: 56 vs. 42%) in Taiwanese children, similar to the trend (76 vs. 53%) reported in a parallel pediatric study in USA (29). Oral sumatriptan has been reported to be not significantly superior to placebo in a trial on migraine in Finland children (30). However, it was effective in 46% of Taiwanese patients here who were refractory to acetaminophen or NSAIDs, although the placebo effect was not examined in the present study. The incidences of adverse effects observed in this study are similar to those of previous reports: none in acetaminophen, higher in NSAIDs, and highest in sumatriptan. It is therefore reasonable to prescribe them as first-, second-, and third-line therapy in order.

For preventive therapy, cyproheptadine, an anti-histamine with anti-serotonergic properties is often used in children younger than 10 year old as a first-line choice for migraine headache (31). Only one-third of patients here responded to cyproheptadine at 2 weeks of therapy although there was no adverse effect observed. Among the other first-line preventive therapies, the short-term response rate to topiramate (70%) was higher than that of flunarizine (55%). This is different from the comparable response rates (81 vs. 80%) observed at 3 months of treatment in a Korean study (32). It suggests that topiramate is more efficient to achieve short-term responsiveness than flunarizine although the long-term effectiveness is comparable to flunarizine (32). The incidences of adverse effects are similar (10%) in both drug groups in the present study whereas more adverse effects were noted in topiramate than flunarizine (10 vs. 5%) in the Korean study (32). The discrepancy may be attributed to differences in race and age of the collected population.

Among the second-line preventive therapies, including valproic acid, propranolol, and gabapentin, the response rate of valproic acid, which has been approved by the United States Food and Drug Administration for migraine prevention for adults (33), is the highest (75%). This is similar to the response rate in an open-label study (34). However, a half of patients had side effects, which is also the highest among the preventive drugs in this study. The effectiveness of beta-blockers for pediatric migraine remains controversial (35–37). This study shows that propranolol has 50% responsiveness and no side effects, which together with the results of valproic acid, is in agreement with the findings of Bidabadi and Mashouf (38) in pediatric migraine prophylaxis. The effectiveness of gabapentin in pediatric migraine has rarely been studied (33, 39). The present study shows a similar responsiveness (50%) to gabapentin as to propranolol although the adverse effects in gabapentin are relatively higher. Interestingly, the use of gabapentin was found to be ineffective in adult migraine treatment (40).

The present study showed that plasma CGRP can significantly differentiate children with migraine from non-headache controls as well as from non-migraine headache. The former, but not the latter, finding is similar to our previous study (19) which revealed that plasma CGRP levels between attacks in the migraineurs were not different from those in non-migraine headache patients (19). This discrepancy may be due to that the recruited patients with relatively more severe or frequent migraine headaches who need either acute or preventive therapy in this study than in the previous one, in which some patients did not require treatment. Nonetheless, the present findings match with the observation by Ashina et al. (41), where plasma CGRP levels were increased in adults with migraine, but not headache. However, significant CGRP level increase was observed in patients with pulsating headache (41). It remains to be elucidated whether pulsating-type headache is attributed to CGRP in the peripheral nerve endings at the meningeal tissue.

In the present study, plasma CGRP levels are significantly elevated in the group requiring preventive therapy instead of acute therapy. It is interesting that disability, evaluated by pediatric migraine assessment score (PedMIDAS), tended to be higher in patients with higher CGRP levels (19). Cernuda-Morollón et al. (42) reported that increased CGRP level measured in peripheral blood during interictal periods could be a biomarker helping in the diagnosis of chronic migraine in adults, whose pain pathways become sensitized after repeated episodes of trigeminal activation (43, 44). This suggests that patients with higher plasma CGRP levels may suffer from more frequent migraine attacks, which may cause disability and require preventive therapy. Plasma CGRP may therefore be a potential biomarker not only for determining the severity or chronicity of migraine (19, 42) but also for predicting the need for preventive therapy.

Among the treatment drugs in our study, topiramate was the only medication to which the responders had significantly higher CGRP levels than the non-responders, and may be predicted by the CGRP level to have a probability of good drug response. Using a threshold of 62.57 or 125.97 pg/ml, the sensitivity and specificity of the plasma CGRP level in predicting topiramate responders was 0.86 and 0.67, or 0.71 and 0.83, respectively, compared to 55.1 pg/ml with sensitivity 0.81 and specificity 0.75 in predicting the diagnosis of pediatric migraine in our previous study (19). Topiramate was developed as an anti-epileptic drug and was also found to be effective in migraine prevention (45). Multi-center, randomized, double-blind, placebo-controlled trials established the efficacy of topiramate in significantly reducing the frequency and severity of migraine attacks in adults (46, 47). Topiramate also showed a trend toward improving headaches in several open-label or randomized, double-blind, placebo-controlled studies in children with migraine (48, 49). However, its exact anti-migraine mechanism(s) remain unclear, but blocking peripheral GluR5 kainate receptors may contribute to its inhibition of neurogenic dural vasodilatation (50). Pre-clinical studies in migraine animal models have shown that topiramate inhibited neurogenic dural vasodilation by inhibiting CGRP release from pre-junctional trigeminal neurons induced by noxious inoculation (17, 51). We also found that topiramate inhibited CGRP immunoreactivity in the trigeminal ganglia and dura (23, 24) of rats in a capsaicin-stimulated migraine model (22). Topiramate has been also reported to enhance GABA currents of GABA_A_ receptors, including the α6 subunit-containing GABA_A_ receptors (α6GABA_A_Rs) (52). The α6GABA_A_Rs are highly expressed in trigeminal ganglia (23, 53) and were recently identified to be a novel drug target for migraine treatment (23). It remains to be elucidated whether the α6GABA_A_R-modulating effect of topiramate contributes to its antimigraine activity. The finding that plasma CGRP levels in the topiramate responders are significantly higher than the non-responders provides clinical evidence supporting the notion that the prophylactic effect of topiramate is mediated by a suppression of CGRP release, which is a marker of trigeminal nerve activation (54). Thus, plasma CGRP may be a potential biomarker for guiding the drug of choice for migraine prevention.

Some limitations of this study need to be addressed. First, patients with mild symptoms of headaches often do not seek medical attention. Thus, it is possible that only patients with greater headache intensity are included in this study. Second, the patient number in some of the sub-groups is small, which may cause statistical bias. Third, placebo effects are not controlled in this study. As such, the comparison should be based on the assumption of equal placebo effects in each sub-group. Fourth, specifying the onset and duration of headache on blood sampling is difficult for pediatric patients, especially for those with multiple episodic attacks within a single day. Duration effects, which may cause variations in the attack levels, have not been controlled in this study. Fifth, the present study does not record body mass index (BMI), which might be a confounding factor because topiramate may cause body weight loss in some patients (55) and obesity has been highlighted to be significantly associated with headache and disability in adults and children (56). However, the impact might be very limited due to short-term therapeutic responses evaluated.

In conclusion, the findings in this study suggest that the plasma CGRP level can differentiate migraine from non-migraine headache conditions in pediatric patients. It may also be a useful biomarker for guiding the drug therapy strategy, as the plasma level of CGRP was significantly higher in pediatric migraineurs who required migraine prophylaxis and were topiramate-responsive. Our results also suggest that NSAIDs are better than acetaminophen for acute therapy whereas topiramate is superior to flunarizine in short-term responsiveness for preventive therapy of pediatric migraine. These results are important especially for young children who cannot clearly describe their headache symptoms and may provide new insights into the clinical practice for the diagnosis and treatment of migraine in children.

## Author Contributions

P-CF designed and conducted experiments, analyzed data, and wrote the paper. ML and L-CC contributed to data interpretation and paper writing. P-HK contributed to results discussion and paper writing. S-HC contributed to data analysis and results interpretation.

### Conflict of Interest Statement

The authors declare that the research was conducted in the absence of any commercial or financial relationships that could be construed as a potential conflict of interest.
